# Comparing PBL and TBL: Insights into effectiveness and efficiency in medical education

**DOI:** 10.1016/j.jtumed.2024.11.006

**Published:** 2024-11-25

**Authors:** Rizky A. Pohan, Ririn D. Astuti, Putri B.A. Pohan, Rikas Saputra, Ronal S. Aditya, Nadhila N. Putri, Elfiadi Elfiadi

**Affiliations:** a Department of Islamic Guidance and Counseling, Institut Agama Islam Negeri Langsa, Langsa, Indonesia; b Department of Biology Education, Yayasan Potret Indonesia Sejahtera, Kota Langsa, Aceh, Indonesia; c Department of Science Education, Universitas Negeri Yogyakarta Yogyakarta, Indonesia; d Department of Guidance and Counseling, Universitas Negeri Malang, Malang, Indonesia; e Department of Nursing, Universitas Negeri Malang, Malang, Indonesia; f Department of Economic Science, Universitas Gadjah Mada Yogyakarta, Indonesia; g Department of Early Childhood Islamic Education, Institut Agama Islam Negeri Lhokseumawe, Lhokseumawe, Indonesia

**Keywords:** Difficulty index, Discrimination index, Problem-Based Learning (PBL), Resource efficiency, Student-centered learning, Team-Based Learning (TBL)

*Dear Editor,*

We are writing in response to your recent publication of research comparing Problem-Based Learning (PBL) and Team-Based Learning (TBL) in medical education.^1^ The findings of this study provide valuable insights into the efficacy and practicality of these student-centered learning strategies, particularly in terms of assessment metrics, resource utilization, and institutional adaptability. The study revealed that the difficulty index of multiple-choice questions (MCQs) showed no significant differences between PBL and TBL. Most questions for both methods were within the optimal difficulty range (26%–89 %), ensuring fair and balanced assessments that promote critical thinking and problem-solving skills. Similarly, the discrimination index, which measures the ability of questions to differentiate between high- and low-performing students, was almost identical for both learning strategies.^2^^,^^3^ This suggests that neither method inherently limits or enhances the ability of students to demonstrate their learning outcomes, offering flexibility for institutions to adopt either approach without compromising assessment precision.

The analysis of distractor functionality, or the effectiveness of incorrect options in engaging students meaningfully, further supported the equivalence of PBL and TBL. Both methods exhibited similar patterns with no statistically significant differences, highlighting the adaptability of MCQ design across these pedagogical approaches. However, the study also emphasized differences in resource efficiency. PBL, while fostering in-depth, small-group discussions conducive to collaborative learning, demands higher resource allocation, including faculty time and smaller student-to-tutor ratios.^4^ In contrast, TBL offers a more resource-efficient alternative suitable for institutions with limited capacity or larger student cohorts.^5^ This operational scalability makes TBL particularly advantageous for broader implementation in under-resourced or large-scale programs, while PBL remains ideal for fostering personalized learning in smaller settings.

These findings are globally relevant as medical schools continue to adopt student-centered approaches. They underscore the importance of aligning pedagogical choices with institutional resources and goals to maintain educational effectiveness while optimizing operational feasibility.^6^^,^^7^ Additionally, the results highlight the need for continued refinement in MCQ design and assessment strategies to ensure validity and reliability across diverse teaching methodologies.^8^^,^^9^

In conclusion, this study demonstrates the comparable educational effectiveness of PBL and TBL, while also providing practical considerations for their implementation. It serves as a valuable reference for medical education institutions worldwide, guiding them in balancing educational outcomes with resource availability to ensure the development of competent, reflective, and adaptable healthcare professionals. Thank you for the opportunity to engage with this important research.

## Source of funding

Beasiswa Indonesia Bangkit, Ministry of Religious Affairs & Lembaga Pengelola Dana Pendidikan (LPDP), Ministry of Finance, Republic of Indonesia, ID number: BU04-231-0000093.
